# Supplementary material for: The Role of NCS1 in Immunotherapy and Prognosis of Human Cancer
Source: Biomedicines. 2023 Oct 12;11(10):2765. doi: 10.3390/biomedicines11102765 (PMC10604305; doi:10.3390/biomedicines11102765)
Supplement: Supplementary file 1 [file biomedicines-11-02765-s001.zip › biomedicines-2622478-supplementary.pdf]

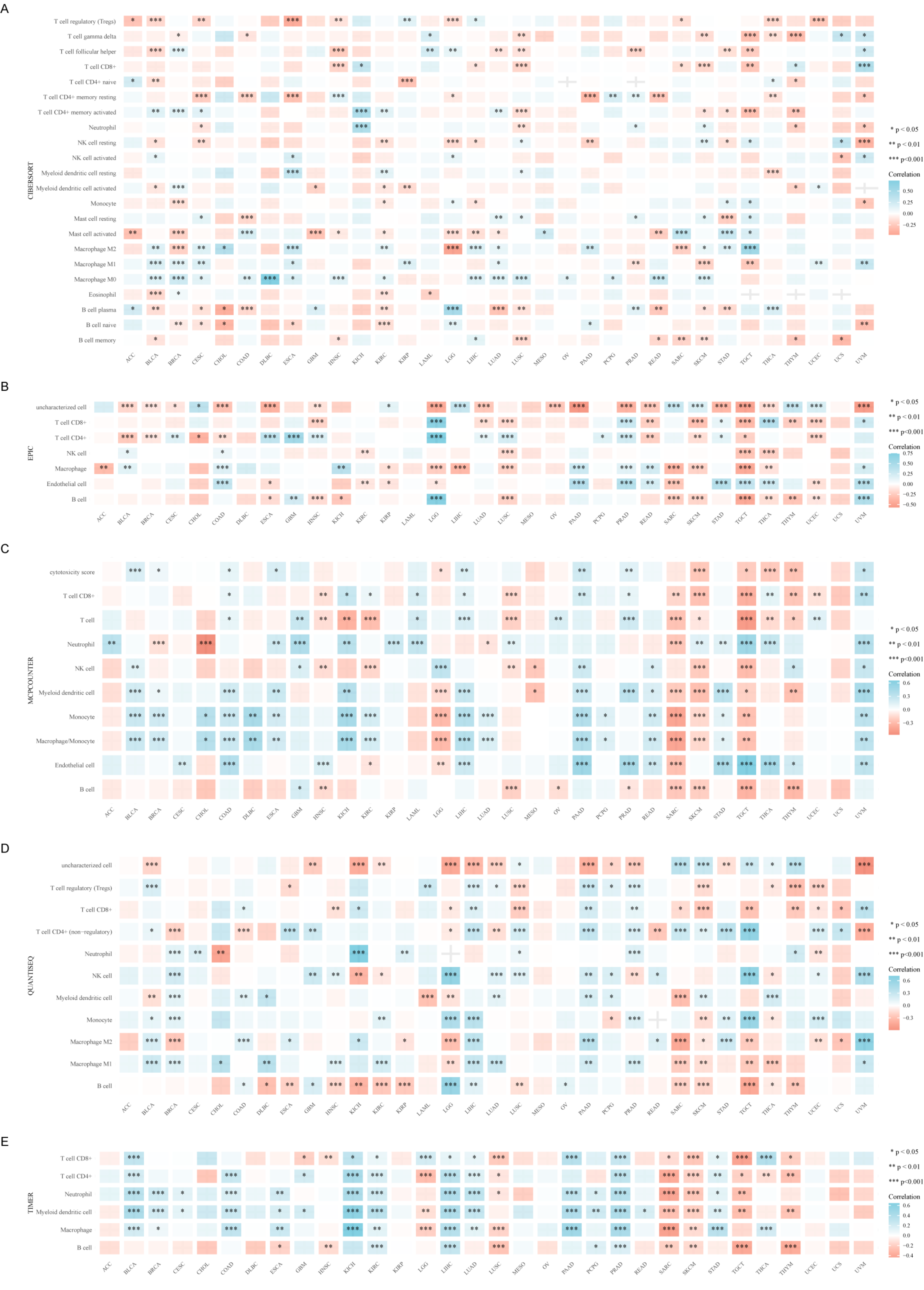
A
CIBERSORT
T cell regulatory (Tregs)
T cell gamma delta
T cell follicular helper
T cell CD8+
T cell CD4+ naive
T cell CD4+ memory resting
T cell CD4+ memory activated
Neutrophil
NK cell resting
NK cell activated
Myeloid dendritic cell resting
Myeloid dendritic cell activated
Monocyte
Mast cell resting
Mast cell activated
Macrophage M2
Macrophage M1
Macrophage M0
Eosinophil
B cell plasma
B cell naive
B cell memory
ACC BLCA BRCA CESC CHOL COAD DLBC ESCA GBM HNSC KICH KIRC KIRP LAML LGG LIHC LUAD LUSC MESO OV PAAD PCPG PRAD READ SARC SKCM STAD TGCT THCA THYM UCEC UCS UVM
* p < 0.05
** p < 0.01
*** p<0.001
Correlation
0.50
0.25
0.00
−0.25
B
EPIC
uncharacterized cell
T cell CD8+
T cell CD4+
NK cell
Macrophage
Endothelial cell
B cell
ACC BLCA BRCA CESC CHOL COAD DLBC ESCA GBM HNSC KICH KIRC KIRP LAML LGG LIHC LUAD LUSC MESO OV PAAD PCPG PRAD READ SARC SKCM STAD TGCT THCA THYM UCEC UCS UVM
* p < 0.05
** p < 0.01
*** p<0.001
Correlation
0.75
0.50
0.25
0.00
−0.25
−0.50
C
MCPCOUNTER
cytotoxicity score
T cell CD8+
T cell
Neutrophil
NK cell
Myeloid dendritic cell
Monocyte
Macrophage/Monocyte
Endothelial cell
B cell
ACC BLCA BRCA CESC CHOL COAD DLBC ESCA GBM HNSC KICH KIRC KIRP LAML LGG LIHC LUAD LUSC MESO OV PAAD PCPG PRAD READ SARC SKCM STAD TGCT THCA THYM UCEC UCS UVM
* p < 0.05
** p < 0.01
*** p<0.001
Correlation
0.6
0.3
0.0
−0.3
D
QUANTISEQ
uncharacterized cell
T cell regulatory (Tregs)
T cell CD8+
T cell CD4+ (non−regulatory)
Neutrophil
NK cell
Myeloid dendritic cell
Monocyte
Macrophage M2
Macrophage M1
B cell
ACC BLCA BRCA CESC CHOL COAD DLBC ESCA GBM HNSC KICH KIRC KIRP LAML LGG LIHC LUAD LUSC MESO OV PAAD PCPG PRAD READ SARC SKCM STAD TGCT THCA THYM UCEC UCS UVM
* p < 0.05
** p < 0.01
*** p<0.001
Correlation
0.6
0.3
0.0
−0.3
E
TIMER
T cell CD8+
T cell CD4+
Neutrophil
Myeloid dendritic cell
Macrophage
B cell
ACC BLCA BRCA CESC CHOL COAD DLBC ESCA GBM HNSC KICH KIRC KIRP LGG LIHC LUAD LUSC MESO OV PAAD PCPG PRAD READ SARC SKCM STAD TGCT THCA THYM UCEC UCS UVM
* p < 0.05
** p < 0.01
*** p<0.001
Correlation
0.6
0.4
0.2
0.0
−0.2
−0.4

A

B

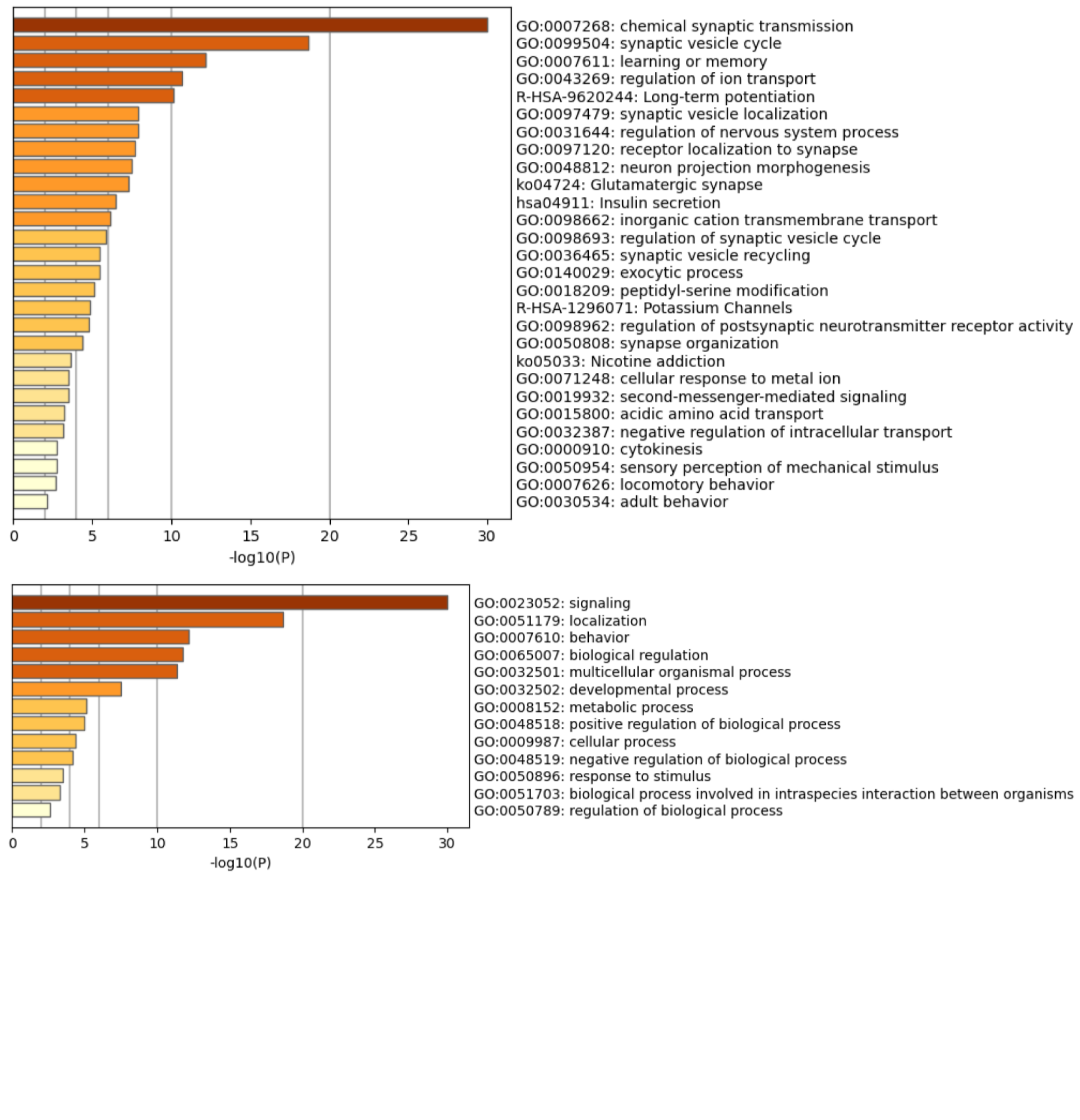
GO:0007268: chemical synaptic transmission
GO:0099504: synaptic vesicle cycle
GO:0007611: learning or memory
GO:0043269: regulation of ion transport
R-HSA-9620244: Long-term potentiation
GO:0097479: synaptic vesicle localization
GO:0031644: regulation of nervous system process
GO:0097120: receptor localization to synapse
GO:0048812: neuron projection morphogenesis
ko04724: Glutamatergic synapse
hsa04911: Insulin secretion
GO:0098662: inorganic cation transmembrane transport
GO:0098693: regulation of synaptic vesicle cycle
GO:0036465: synaptic vesicle recycling
GO:0140029: exocytic process
GO:0018209: peptidyl-serine modification
R-HSA-1296071: Potassium Channels
GO:0098962: regulation of postsynaptic neurotransmitter receptor activity
GO:0050808: synapse organization
ko05033: Nicotine addiction
GO:0071248: cellular response to metal ion
GO:0019932: second-messenger-mediated signaling
GO:0015800: acidic amino acid transport
GO:0032387: negative regulation of intracellular transport
GO:0000910: cytokinesis
GO:0050954: sensory perception of mechanical stimulus
GO:0007626: locomotory behavior
GO:0030534: adult behavior
0 5 10 15 20 25 30
-log10(P)
GO:0023052: signaling
GO:0051179: localization
GO:0007610: behavior
GO:0065007: biological regulation
GO:0032501: multicellular organismal process
GO:0032502: developmental process
GO:0008152: metabolic process
GO:0048518: positive regulation of biological process
GO:0009987: cellular process
GO:0048519: negative regulation of biological process
GO:0050896: response to stimulus
GO:0051703: biological process involved in intraspecies interaction between organisms
GO:0050789: regulation of biological process
0 5 10 15 20 25 30
-log10(P)

A

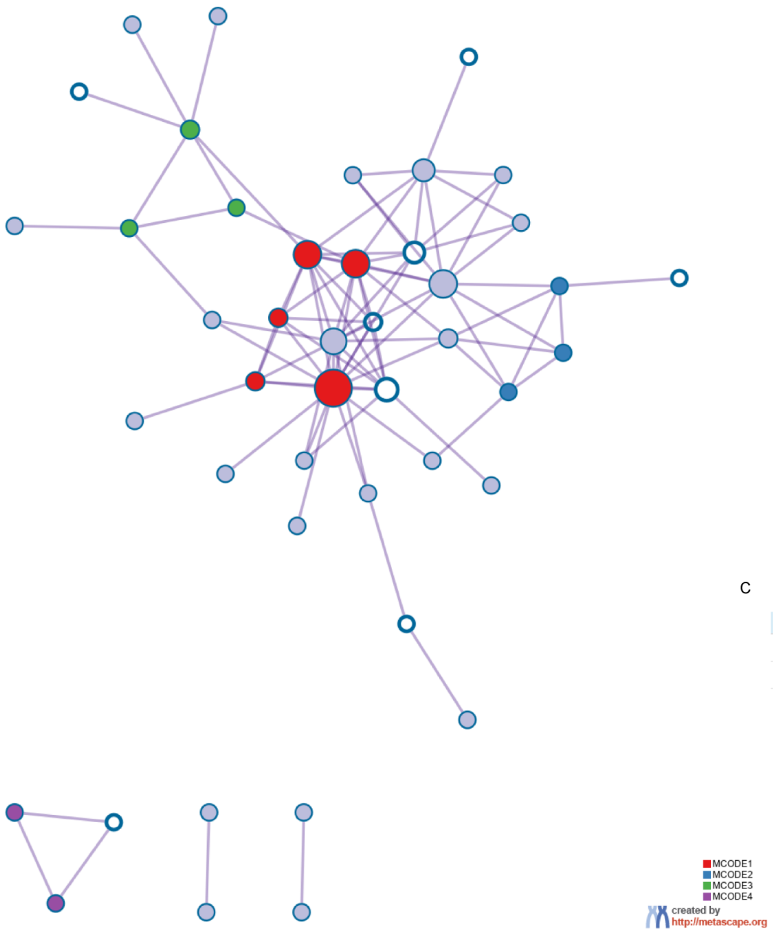

B

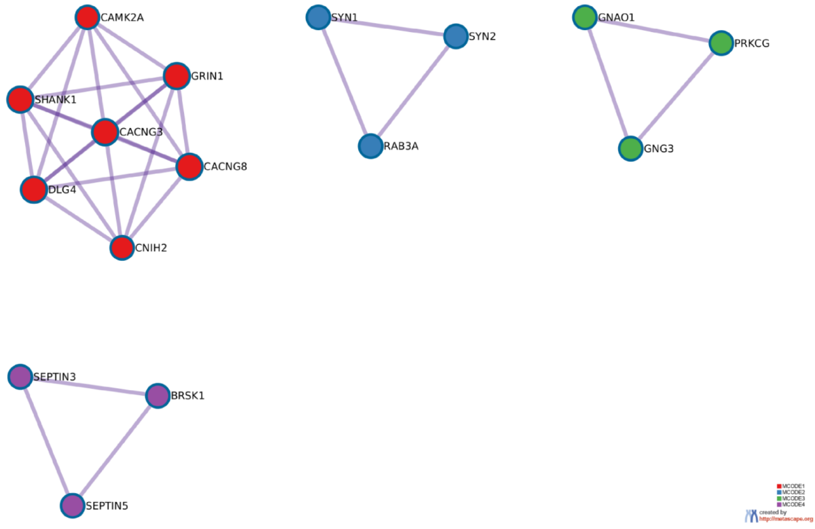

C

GO	Description	Log10(P)
GO:0007268	chemical synaptic transmission	-28.0
GO:0098916	anterograde trans-synaptic signaling	-28.0
GO:0099537	trans-synaptic signaling	-27.9

Color	MCODE	GO	Description	Log10(P)
red	MCODE_1	GO:0050804	modulation of chemical synaptic transmission	-12.7
red	MCODE_1	GO:0099177	regulation of trans-synaptic signaling	-12.7
red	MCODE_1	GO:0099601	regulation of neurotransmitter receptor activity	-12.0
blue	MCODE_2	GO:0097091	synaptic vesicle clustering	-10.0
blue	MCODE_2	R-HSA-181429	Serotonin Neurotransmitter Release Cycle	-9.7
blue	MCODE_2	R-HSA-212676	Dopamine Neurotransmitter Release Cycle	-9.3
green	MCODE_3	ko04727	GABAergic synapse	-7.5
green	MCODE_3	hsa04727	GABAergic synapse	-7.5
green	MCODE_3	ko05032	Morphine addiction	-7.5

Table S1. The TPM expression of NCS1 in 32 kinds of tumors from TCGA database.

kinds	tumor	normal
ACC	16.2	17.94
BLCA	12.85	34.517
BRCA	12.85	34.517
CESC	N/A	
CHOL	2.17	0.18
COAD	8.39	59.738
DLBC	2.17	0.18
ESCA	N/A	
GBM	28.249	222.12
HNSC	27.04	12.505
KICH	0.53	15.87
KIRC	6.19	15.42
KIRP	N/A	
LAML	0.54	12.64
LGG	35.465	222.12
LIHC	1.53	0.735
LUAD	N/A	
LUSC	18.075	6.47
MESO	N/A	
OV	16.505	7.71
PAAD	8.87	1.6
PCPG	8.87	1.6
PRAD	11.775	26.479
READ	7.91	65.771
SARC	7.91	65.771
SKCM	18.47	13.32
STAD	N/A	
TGCT	8.18	3.56
THCA	16.755	32.3
THYM	9.407	0.18
UCEC	11.875	42.541
UCS	N/A	

Table S2. The most similar genes to NCS1 from GEPIA 2.0.

Gene Symbol	Gene ID	PCC	Gene Symbol	Gene ID	PCC
PSD	ENSG00000059915.16	0.81	KCNJ4	ENSG00000168135.4	0.71
PACSIN1	ENSG00000124507.10	0.81	PHF24	ENSG00000122733.12	0.71
JPH3	ENSG00000154118.12	0.81	RP11-503P10.1	ENSG00000272056.1	0.71
KCNN1	ENSG00000105642.15	0.8	AC005330.2	ENSG00000267372.2	0.71
CAMK2A	ENSG00000070808.15	0.8	RIMS3	ENSG00000117016.9	0.71
KCNK4	ENSG00000182450.12	0.8	SYN2	ENSG00000157152.16	0.71
SNPH	ENSG00000101298.13	0.8	ADGRB2	ENSG00000121753.12	0.71
SEPT5	ENSG00000184702.17	0.79	GNAO1	ENSG00000087258.13	0.71
DLGAP3	ENSG00000116544.11	0.78	CAMKV	ENSG00000164076.16	0.71
BRSK1	ENSG00000160469.16	0.77	C1orf95	ENSG00000203685.9	0.7
CASKIN1	ENSG00000167971.15	0.77	CA11	ENSG00000063180.8	0.7
MAP1A	ENSG00000166963.12	0.77	3-Sep	ENSG00000100167.19	0.7
IDS	ENSG00000010404.17	0.77	RP11-826N14.2	ENSG00000248469.1	0.7
RP11-1263C18.1	ENSG00000273238.1	0.77	SLC17A7	ENSG00000104888.9	0.7
CHN1	ENSG00000128656.13	0.77	FAM131A	ENSG00000175182.13	0.7
MAP3K10	ENSG00000130758.7	0.77	NRGN	ENSG00000154146.12	0.7
HSD11B1L	ENSG00000167733.13	0.76	JPH4	ENSG00000092051.16	0.7
CACNG3	ENSG00000006116.3	0.76	CALM3	ENSG00000160014.16	0.7
PDXP	ENSG00000241360.1	0.76	NAPB	ENSG00000125814.17	0.7
OLFM1	ENSG00000130558.18	0.76	CCK	ENSG00000187094.11	0.7
GNG3	ENSG00000162188.5	0.75	MGAT5B	ENSG00000167889.12	0.7
RP11-320H14.1	ENSG00000260878.1	0.75	FBXO41	ENSG00000163013.11	0.69
DLG4	ENSG00000132535.18	0.75	LLNLF-187D8.1	ENSG00000277531.1	0.69
KCNH3	ENSG00000135519.6	0.75	C11orf87	ENSG00000185742.6	0.69
PRKAR1B	ENSG00000188191.14	0.74	PIANP	ENSG00000139200.13	0.69
RP13-514E23.1	ENSG00000261496.1	0.74	SNX32	ENSG00000172803.17	0.69
PRKCG	ENSG00000126583.10	0.74	CABP1	ENSG00000157782.9	0.69
RAB3A	ENSG00000105649.9	0.74	HABP4	ENSG00000130956.13	0.69
CACNG8	ENSG00000142408.2	0.74	PNMAL2	ENSG00000204851.6	0.69
FXYD7	ENSG00000221946.7	0.74	CNIH2	ENSG00000174871.10	0.69
SLC25A23	ENSG00000125648.14	0.74	RP11-586D19.1	ENSG00000249896.1	0.69
SYN1	ENSG00000008056.12	0.74	ATL1	ENSG00000198513.11	0.69
PDZD4	ENSG00000067840.12	0.74	RP5-896L10.1	ENSG00000232825.1	0.69
DMTN	ENSG00000158856.17	0.73	TAGLN3	ENSG00000144834.12	0.69
GRIN1	ENSG00000176884.14	0.73	RNF208	ENSG00000212864.3	0.69
SULT4A1	ENSG00000130540.13	0.73	CEND1	ENSG00000184524.5	0.68
FBXL16	ENSG00000127585.11	0.72	MAGEE1	ENSG00000198934.4	0.68
KIF5A	ENSG00000155980.11	0.72	SNCB	ENSG00000074317.10	0.68
TPPP	ENSG00000171368.11	0.72	MIR124-2HG	ENSG00000254377.5	0.68
SHANK1	ENSG00000161681.15	0.72	RUNDC3A	ENSG00000108309.12	0.68
FRMPD4	ENSG00000169933.12	0.72	RP11-317N8.3	ENSG00000257272.1	0.68
RP11-416I2.1	ENSG00000260328.1	0.72	RP11-389G6.3	ENSG00000261292.2	0.68
C1orf216	ENSG00000142686.7	0.72	CPNE6	ENSG00000100884.9	0.68
NECAB1	ENSG00000123119.11	0.72	LINC00950	ENSG00000281205.1	0.68
DLG2	ENSG00000150672.16	0.72	SERP2	ENSG00000151778.10	0.68

RAPGEF4	ENSG00000091428.17	0.72	GABRA5	ENSG00000186297.11	0.68
APBA1	ENSG00000107282.7	0.72	RP11-713C5.1	ENSG00000265579.1	0.68
NPTXR	ENSG00000221890.2	0.72	RP11-43A14.2	ENSG00000282375.1	0.68
CELF5	ENSG00000161082.12	0.71	CAMK1G	ENSG00000008118.9	0.68
THSD4-AS1	ENSG00000259964.6	0.71	CTD-2228A4.1	ENSG00000271892.1	0.68

PCC: Pearson correlation coefficient
